# Psychometric Adequacy of Recovery Enhancing Environment (REE) Measure: CHIME Framework as a Theory Base for a Recovery Measure

**DOI:** 10.3389/fpsyt.2020.00595

**Published:** 2020-06-30

**Authors:** Patricia Penas, Jose Juan Uriarte, Susana Gorbeña, Maria Concepcion Moreno-Calvete, Priscilla Ridgway, Ioseba Iraurgi

**Affiliations:** ^1^ Department of Personality, Assessment and Psychological Treatment, University of Deusto, Bilbao, Spain; ^2^ Red de Salud Mental, Biocruces Bizkaia Health Research Institute, Basque Health Service, Bizkaia Mental Health Network, Bilbao, Spain; ^3^ Independent Consultant, Houston, TX, United States

**Keywords:** personal recovery, recovery enhancing environment, REE, assessing personal recovery, CHIME framework, psychometric properties, severe mental illness

## Abstract

**Purpose:**

The aim of this study was to assess to what extent the recovery elements of the Recovery Enhancing Environment (REE) instrument measured the dimensions proposed by the CHIME framework, (Connectedness, Hope and optimism about future, Identity, Meaning in life and Empowerment dimensions), so as to evaluate personal recovery in people with severe mental illness.

**Methods:**

Two processes were conducted. Firstly, five experts matched the elements of recovery evaluated by the REE items with the CHIME domains and subdomains. Then, the resulting structure from those experts agreement was analyzed with different confirmatory factor analyses (CFA) using responses to the recovery elements dimension of the REE of 312 mental health service users.

**Results:**

The percentage of agreements and the kappa coefficients were adequate taking into account the CHIME dimensions (κ = 0.57 to 0.69, total κ = 0.74); however, lower agreement was found at the subdimensions level. Some indexes of the CFA were acceptable for a second order factor analysis [*χ* ^2^_(242)_= 346.03, *p* < 0.001, CFI= 0.931, RMSEA= 0.037 (0.028 to 0.046)] and the most adequate solution was obtained from the bi-factorial structure (*χ* ^2^_(223)_=233.19, *p*=0.306, CFI= 0.993, RMSEA= 0.012 [0.000 to 0.027]).

**Conclusions:**

Despite the subjective and complex nature of the personal recovery construct, the REE measure can be a valid instrument to verify the existing CHIME conceptual framework, since two of the models tested have resulted in adequate indexes and were also congruent with the theoretical framework and the statistical solution. Thus, REE can be used to obtain a global index of Personal Recovery dimension, and the five indicators proposed by the CHIME framework.

## Introduction

Personal recovery of people suffering from severe mental disorders (SMD) is receiving increasing attention from practitioners and mental health policy makers (1). The concept of Personal recovery, defined by Anthony (2) as “a way of living a satisfying, hopeful and contributing life, even with the limitations caused by the illness”(p. 15) has been widely accepted. However, given its complex and subjective nature (3), its definition and boundaries have not been clearly defined, an issue that could hinder the selection of the most appropriate instruments for its evaluation (4).

In recent years, progress has been made with regards to the personal recovery conceptual framework. The approach proposed by Leamy, Bird, Le Boutillier, Williams and Slade, summarized using the acronym CHIME (5), has been gaining importance (6, 7). This conceptual framework is composed by five recovery processes: Connectedness, Hope and optimism about the future, Identity, Meaning in life, and Empowerment. The study conducted by Slade el al. has provided some international validity (8), and the work of Bird et al. has validated the framework through deductive and inductive analyses with individuals using mental health services (9).

There are different tools available for evaluating personal recovery, with different elements, such as the Recovery Assessment Scale (RAS) (10) to assess personal recovery, or the Recovery Self-Assessment (RSA) (11) to evaluate services with regards to recovery orientation, but those instruments do not fit appropriately with the five processes defined in the CHIME framework (4, 12). However, as it is highlighted in the review about measures to assess recovery orientation of mental health services made by Williams and his colleagues (4), the Recovery Enhancing Environment (REE) (13) is the measure that most closely could match the CHIME framework. REE is an instrument developed through content analysis of mental health users´ experiences about their recovery, and the perceived support received during this process, with the aim of assessing personal recovery (13).

Thus, the aim of this study is to assess to what extent the Recovery Enhancing Environment instrument measures the dimensions of the CHIME framework. Firstly, the model will be tested using an expert judgment approach, which implies a qualitative methodology. Subsequently, the resulting structure will be analyzed with a quantitative methodology in order to assess how do the responses of the mental health users accommodate to the proposed model.

## Method

### Participants

Participants were selected from the Severe Mental Disorders Program of the Biscay Mental Health Services Network, Spain. Almost 2,000 patients with a diagnosis of severe mental chronic illness (mainly schizophrenia) receive services, including specific treatment planning and regular assessment.

Three hundred twelve patients were randomly chosen out of the 1949 registered participants. The sample was stratified according to gender, age and type of mental health service used (outpatient, daycare hospital, assertive community treatment, and psychiatric hospital) with an estimation error of 5.1% (at the 95% confidence level). Inclusion criteria were being 18 years or older, and being an active member of the program. Exclusion criteria were the absence of informed consent of the patient or legal guardian, language difficulties or communication problems, and presence of significant clinical symptoms that prevented participation such as acute hospitalization.

In terms of demographic and clinical characteristics, 189 participants were males and 123 females, the majority were single with an average age of 49.17 years (SD= 10.97) and living with their families. One hundred ninety-four individuals (62.2%) were treated in outpatient mental health centers, 75 (24%) in daycare hospitals, 22 (7.1%) in assertive community treatment, and 21 (6.7%) in hospital settings. The 56.1% of the patients were diagnosed with schizophrenia disorder, 12% had a bipolar disorder, and 8.9% a schizoaffective disorder. The average number of years in treatment was 17.37 (SD= 8.70), with a range between 1 and 45 years. During the evolution of the disorder, 75.6% of the participants had required some hospitalization (the mean of episodes= 6.65, SD= 8.34). **Table 1** presents sociodemographic characteristics by type of clinical resource utilized.

**Table 1:** Descriptive statistics and contrast tests of the sociodemographic variables in relation to type of care center.

	Total (N=312)		OMHC (N=194)		DH (N=75)		ACT (N=22)		HS (N=21)		F	p
	M	SD	M	SD	M	SD	M	SD	M	SD		
**Age**	49.17	10.97	50.71	10.99	46.85	8.80	50.95	12.24	41.43	12.35	5.546	0.001
	**n**	**%**	**n**	**%**	**n**	**%**	**n**	**%**	**n**	**%**	**χ²**	**p**
**Gender**												
Male	189	60.6	114	58.8	47	62.7	14	63.6	14	66.7	0.81	0.845
Female	123	39.4	80	41.2	28	37.3	8	36.4	7	33.3		
**Marital status**												
Single	215	68.9	124	63.9	59	78.7	15	68.2	17	81.0	12.84	0.170
Married	40	12.8	33	17.0	3	4.0	2	9.1	2	9.5		
Divorced/separated/widower	50	16.0	31	16.0	12	16.0	5	22.7	2	9.5		
Others	7	2.2	6	3.1	1	1.3	0	0.0	0	0.0		
**Studies**												
Up to secondary education	206	66.0	131	67.5	48	64.0	15	68.2	12	57.1	14.35	0.026
Professional training	69	22.1	36	18.6	23	30.7	2	9.1	8	38.1		
University studies	36	11.5	23	13.4	4	5.3	5	22.7	1	4.8		
**Employment status**												
Working	50	16.0	37	19.1	6	8.0	3	13.6	4	19.0	15.31	0.083
Unemployed	56	17.9	30	15.5	12	16.0	6	27.3	8	38.1		
Long term disability	137	43.9	81	41.8	41	54.7	9	40.9	6	28.6		
Others*	69	22.1	46	23.7	16	21.3	4	18.2	3	14.3		
**Work before the disorder**												
Yes	248	79.5	160	82.5	54	72.0	16	72.7	18	85.7	6.74	0.081
No	54	17.3	26	13.4	19	25.3	6	27.3	3	14.3		
Miss	10	3.2	8	4.1	2	2.7	0	0.0	0	0.0		
**Living situation**												
Family	204	65.7	132	68.0	45	60.0	14	63.6	13	61.9	8.29	0.505
Autonomous	70	22.4	42	21.8	18	24.0	7	31.8	3	14.3		
Supported Housing	23	7.4	12	6.2	8	10.7	0	0.0	3	14.3		
Others	14	4.5	7	3.6	4	5.3	1	4.5	2	9.5		

OMHC, Outpatient Mental Health Centers; DH, Day Hospitals; ACT, Assertive Community Treatment; HS, Hospital Settings; *Others in employment statues include students (n=14), housework (n=26), retired (n=11) and others (n=18).

### Instrument

The Recovery Enhancing Environment measure (REE) (13), also known as DREEM in the United Kingdom, is an instrument composed by four sections: importance of recovery elements, experience of recovery elements, organizational climate, and recovery markers. For this study, only the first section was used, where the relative importance the service users give to each of the 24 elements related to recovery is evaluated. Those 24 items cover elements such as “*having a sense of meaning in life is important to my recovery*” or “*having hope is important to my recovery*”. They are scored with a five-point Likert scale (4= strongly agree to 0= strongly disagree), with higher scores indicating greater importance. The Spanish version was completed. This was adapted and validated by Uriarte, Penas, Moreno-Calvete, Ridgway and Iraurgi, (14), who have reported adequate convergent and construct validity. The internal consistency of this section in the original questionnaire was 0.94 and in the current study, it has resulted in a Cronbach alpha of 0.90.

### Procedure

Participants were randomly selected from a stratified sample. Interviews were conducted in order to explain the study and sign the informed consent. If the participant did not satisfy the inclusion criteria or refused participation, it was replaced by another patient selected randomly. As part of an ongoing strategy of patient empowerment, four patients with personal experience in the recovery process were hired to perform the data collection. For this purpose, they received training about the concept of recovery, the utilization of the REE instrument and interviewing skills, including aspects such as confidentiality. The present project received the approval of the Clinical Research Ethics Committee of the Health Services of the Basque Country.

Subsequently, three clinicians and two researchers coded each element evaluated through the REE instrument according to the CHIME domains and subdomains proposed by Leamy et al. (5). All the raters had clinical and research expertise of more than 5 years.

### Statistical Analyses

In order to explore the factorial structure that was going to be tested through confirmatory factor analyses (CFA), the percentage of judges’ agreement (%) for each item in the five CHIME domains was calculated. This agreement percentage indicated how often the experts rated the same domain of the CHIME for the same items of the REE. An 80% of percentage agreement was considered adequate (15). Besides, the reliability of the items using kappa coefficients was calculated, where a kappa value below 0.40 was considered poor agreement, values from 0.40 to 0.75 were moderate to good, and above 0.75 was excellent (16).

Having the conceptual structure defined, four different types of CFA models were tested: unidimensional, five independent correlated factors, a second order factor structure, and a bi-factorial model. The EQS (6.1) program was used (17). Correlation matrix, multivariate skewness and kurtosis were evaluated with Mardia´s test, and in the event of deviation from normality (Mardia´s coefficient >5), the Weighted Least Square (WLS) estimation method was used with the robust methods proposed by Satorra and Bentler (18). The following indicators were used to assess the level of goodness of fit: the chi-squared test (*χ* ^2^) to assess the probability that the variation between the sampling variance and covariance matrix and the matrix resulting from the hypothesized model was random; Normed Chi-square (*χ* ^2^/df) whose values should be between 1 and 3; the Akaike Information Criterion (AIC); the Comparative Fit Index (CFI) in which values should be >0.90; the Bentler Bonett Normed Fit Index (BB-NFI) and Bentler Bonnett Non-normed Fit Index (BB-NNFI); and finally, the Root Mean Square Error of Approximation (RMSEA) where a value <0.05 is considered adequate and <0.08 acceptable, with a 90% confidence interval.

Provided that the bi-factor models offered a superior fit than the other models, the method proposed by Rodriguez, Reise and Haviland (19, 20) was used to test the specification and quality of the measurement model, as well as the quality of unit-weighted total and subscales score composite of CHIME model from the REE instrument. The following statistical indices were calculated: Omega reliability coefficients, construct reliability (index H), explained common variance (ECV) and percentage of uncontaminated correlation (PUC).

The Omega indices are a family of reliability indicators based on the factor loadings of a specific structural model. It is similar to alpha coefficient, so its values also range from 0 (no reliability) to 1 (perfect reliability) (20). There are different omega values for the bifactor CFA model, depending on what is being estimated. As it is explained by Osborne and his collages (21), the Omega (*ω*) coefficient is the proportion of variance of the compound score for the total scale that is attributable to all sources of common variance; this Omega coefficient is calculated also for the scores given in each subscale (Omega subscale, *ω*_s_). The hierarchical forms of Omega represent the proportion of variance in total scores that is explained by the general factor (Omega hierarchical, *ω*_H_), and the variance in the compose score explained by the subscale, dividing the variance explained by the general factor (Omega hierarchical subscale, *ω*_HS_). Consequently, the Omega coefficients are indicating multidimensional reliability, whereas the hierarchical ones estimate unidimensionality (22).

Moreover, the Hancock and Muller index (H) was calculated to evaluate the construct reliability (19). H values provide the correlation between a factor and an optimally weighted item composite. When the H is high (>0.70), the latent variable is well defined by the indicators (23). Explained common variance (ECV) can be used to judge the essential unidimensionality of the common variance in an item set. Higher ECV values indicate a strong general factor, which may guide the decision to fit a unidimensional model even with data that are multidimensional (20). Finally, the percent of uncontaminated correlation (PUC), in conjunction with ECV, are an indicator of the possible biasing effects of forcing multidimensional data into a unidimensional structure (24, 25).

## Results

**Table 2** presents percentages of agreement and kappa coefficients. Raters’ column indicates the number of raters that agreed, followed by a capital letter and a lower case letter. The capital letter indicates the CHIME dimension selected by the rater (C—Connectedness, H—Hope, I—Identity, M—Meaning and E—Empowerment). The lowercase letter refers to the subdimension to which the item was assigned. For instance, in item 9, the five raters agreed it belonged to the Connectedness dimension (C) and the peer support and support groups subdimension (a). In the item 12 (1-Ca, 3Cb, 1Ha), the first rater has matched this item to “Connectedness” (C) and to the “a” subdimension inside connectedness (“peer support and support groups” (a). Other 3 raters raters evaluated this item also as part of “Connectedness” (C), but then they have selected the subdimension “b” (“relationships”). Finally, the last rater has matched it with the dimension of “Hope” (H) in its subdimension “a” of (“belief in the possibility of recovery”). As it can been seen in **Table 2**, in 14 out of 24 elements, there was a perfect agreement between experts (100%) when assigning each REE item to one of the five CHIME domains, in six items it reached 80%, and four elements have a 60% of agreement. However, there is some variability in the judgments made by the different raters in relation to the subdimensions of the conceptual framework. For the total framework, a good kappa coefficient of 0.74 was obtained, also acceptable for the five dimensions (all of them ranging from 0.69 to 0.57).

**Table 2:** CHIME conceptual framework, percentage agreement and kappa coefficients of the REE instruments item.

CHIME Conceptual Framework [from (5)]		Item	Statement	Raters*	Agreement	Kappa
**Connectedness (C)**		9	Mutual self-help/peer support…	5-Ca	100	
a	Peer support and support groups	11	Being involved in, and a part of, the larger community…	5-Cd	100	0.69
b	Relationships	12	Having positive relationships…	1-Ca, 3-Cb, 1Ha	80	
c	Support from others	22	Having assistance when I am in crisis…	1-Ca, 1-Cb, 3-Cc	100	
d	Being part of the community	24	Having helpers who really care about me and my recovery…	1-Cb, 4-Cc	100	
**Hope & Optimism (H)**						
a	Belief in possibility of recovery	3	Having hope…	3-Ha, 1-Hc, 1-Hd	100	
b	Motivation to change	20	Taking on new challenges and moving out of my comfort zone…	1-Hb, 4-He	100	
c	Hope-inspiring relationships	21	Having positive role models…	4-Ha, 1-Hd	100	
d	Positive thinking and valuing success					
e	Having dreams and aspirations					0.59
**Identity (I)**						
a	Dimensions of identity	1	Having a positive sense of personal identity beyond my psychiatric disorder….	3-Ia, 2-Ib	100	
b	Rebuilding/redefining positive sense of identity	8	Having my rights respected and upheld…	3-Ia, 2-Eb	60	
c	Overcoming stigma	19	Challenging stigma and discrimination…	5-Ic	100	
		23	Intimacy and sexuality…	4-Ia, 1-Cb	80	0.64
**Meaning & Purpose (M)**						
a	Meaning of mental illness experiences	2	Having a sense of meaning in life…	2-Ma, 1-Md, 1-Mf, 1-Hd	80	
b	Spirituality	10	Being involved in meaningful activities…	1-Md, 2-Me, 2-Mf	100	
c	Quality of life	15	Having my basic needs met…	3-Mc, 1-Ic, 1-Eb	60	
d	Meaningful life and social roles	17	Spirituality…	5-Mb	100	
e	Meaningful life and social goals	18	Taking on, and succeeding in, normal social roles …	4-Mb, 1-Cb	80	
f	Rebuilding life					0.57
**Empowerment (E)**		4	Having up-to-date knowledge about psychiatric disorders & the most effective treatments …	3-Ea, 1-Eb, -1Hc	80	
a	Personal responsibility	5	Being able to self-manage symptoms and avoid relapse…	4-Eb, 1-Ea	100	
b	Control over life	6	Improving my general health and wellness…	1-Ea, 2-Eb, 2-Mc	60	
c	Focusing upon strengths	7	Being an active consumer and directing my own recovery…	3-Ea, 1-Eb, 1-Hc	80	
		13	Identifying and building on my personal strengths…	5-Ec	100	
		14	Developing new skills…	3-Ec, 2-Ib	60	
		16	Having a sense of control over my life and feeling empowered…	5-Eb	100	0.59

All the items finish the sentence with … to my recovery. The kappa coefficient for the total framework is 0.74. *Raters´ column indicates the number of raters that agreed, the following capital letter refers to the CHIME dimension selected by the rater (C-Connectedness, H-Hope, I-Identity, M-Meaning and E-Empowerment) and the last lowercase letter refers to the subdimension to which the item was assigned in the previous dimension.

The model that resulted from the experts agreement was used for conducting the different confirmatory factor analyses (see the model in **Table 2**). Firstly, the five dimensions were tested separately, resulting in an adequate fit of the items to four subscales (Connectedness, Identity, Meaning and purpose, and Empowerment). The Index of Hope and optimism showed a perfect fit, given it is a saturated model with a low number of items. Results are presented in **Table 3**. Four different models were verified through CFA. The most adequate indexes were obtained for the second order factor structure [*χ* ^2^_(242)_= 346.03, *p* < 0.001, AIC= −137.96, CFI= 0.931, BB-NFI= 0.807, BB-NNFI= 0.922, RMSA= 0.037 (0.028 to.046)], and the bi-factorial model, which resulted in a good fit between the data and the conceptual model [*χ* ^2^_(223)_=233.19, *p*=0.306, AIC= −212.80, CFI= 0.993, BB-NFI= 0.870, BB-NNFI= 0.992, RMSA= 0.012 (0.000 to 0.027)]. Note that in both models the BB-NFI index does not reach the standard reference value (> 0.90), but the bi-factoral model is close to this value.

**Table 3:** Structural models.

	χ^2^	p	df	χ^2^/df	AIC	CFI	BB-NFI	BB-NNFI	RMSEA	IL	SL
**Dimensions**											
Connectedness	11.62	0.040	5	2.324	1.624	0.964	0.941	0.929	0.066	0.013	0.116
Hope & Optimism	0	*	0		*	–	1.000	–	–	–	–
Identity	0.37	0.827	2	0.188	−3.622	1.000	0.994	1.08	0.000	0.000	0.066
Meaning & Purpose	2.93	0.710	5	0.586	−7.069	0.999	0.982	0.999	0.000	0.000	0.059
Empowerment	22.17	0.075	14	1.583	−5.829	0.985	0.962	0.978	0.044	0.000	0.076
**Models**											
Unidimensional	381.99	0.001	252	1.515	−122.00	0.941	0.787	0.906	0.041	0.032	0.049
5 correlated factors	373.94	0.001	241	1.545	−110.05	0.913	0.792	0.901	0.042	0.038	0.050
Second order model (5 + 1)	346.03	0.001	242	1.016	−137.96	0.931	0.807	0.922	0.037	0.028	0.046
Bi factor model	233.19	0.306	223	1.045	−212.80	0.993	0.870	0.992	0.012	0.000	0.027

*Nonpositive degrees of freedom, probability computations are undefined. - Difficulties in the calculation due to the number of items. AIC, Akaike Information Criterion; CFI, Comparative Fit Index; BB-NFI, Bentler Bonett Normed Fit Index; BB-NNFI, Bentler Bonett Non-Normed Fit Index; RMSEA, Root Mean Square Error of Approximation; IL, Inferior Limit; SL, Superior Limit.

**Figure 1:**
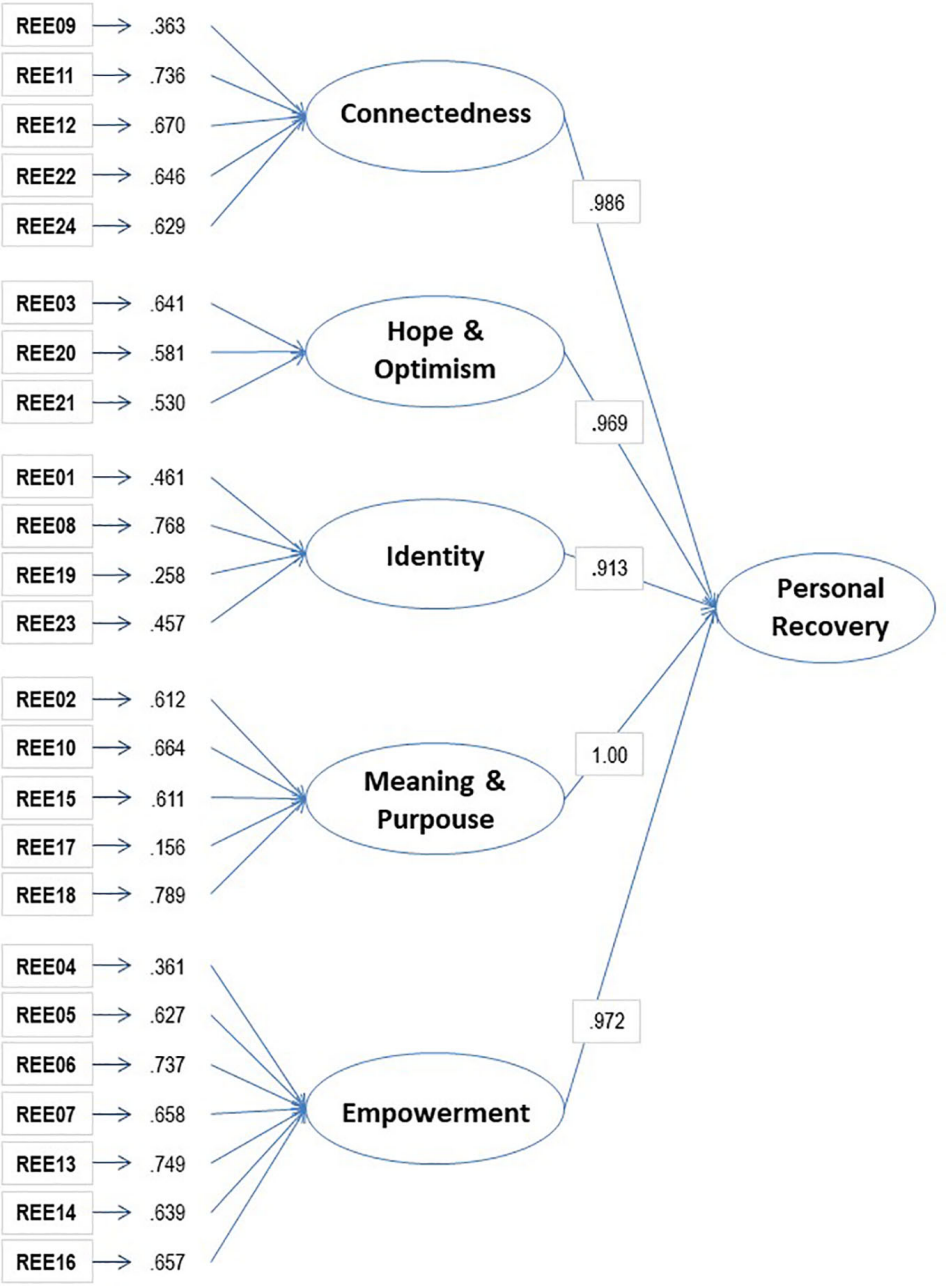
Second order factor structure of the REE.

The second order factor structure presented in **Figure 1** shows that most items highly loaded in the five dimensions (all above 0.361, except I17 and I19). Likewise, those five factors loaded highly (between 0.913 to 1.00) in a second order factor, which is the general factor of personal recovery. Furthermore, the construct reliability and extracted variance were calculated for the five factors, resulting in adequate values for Empowerment (0.79; 35.26%) and Connectedness (0.70; 33.21%) and lower scores for Meaning and purpose (0.67; 31.83%), Hope and optimism (0.56; 29.76%) and Identity (0.53; 24.16%).

**Figure 2:**
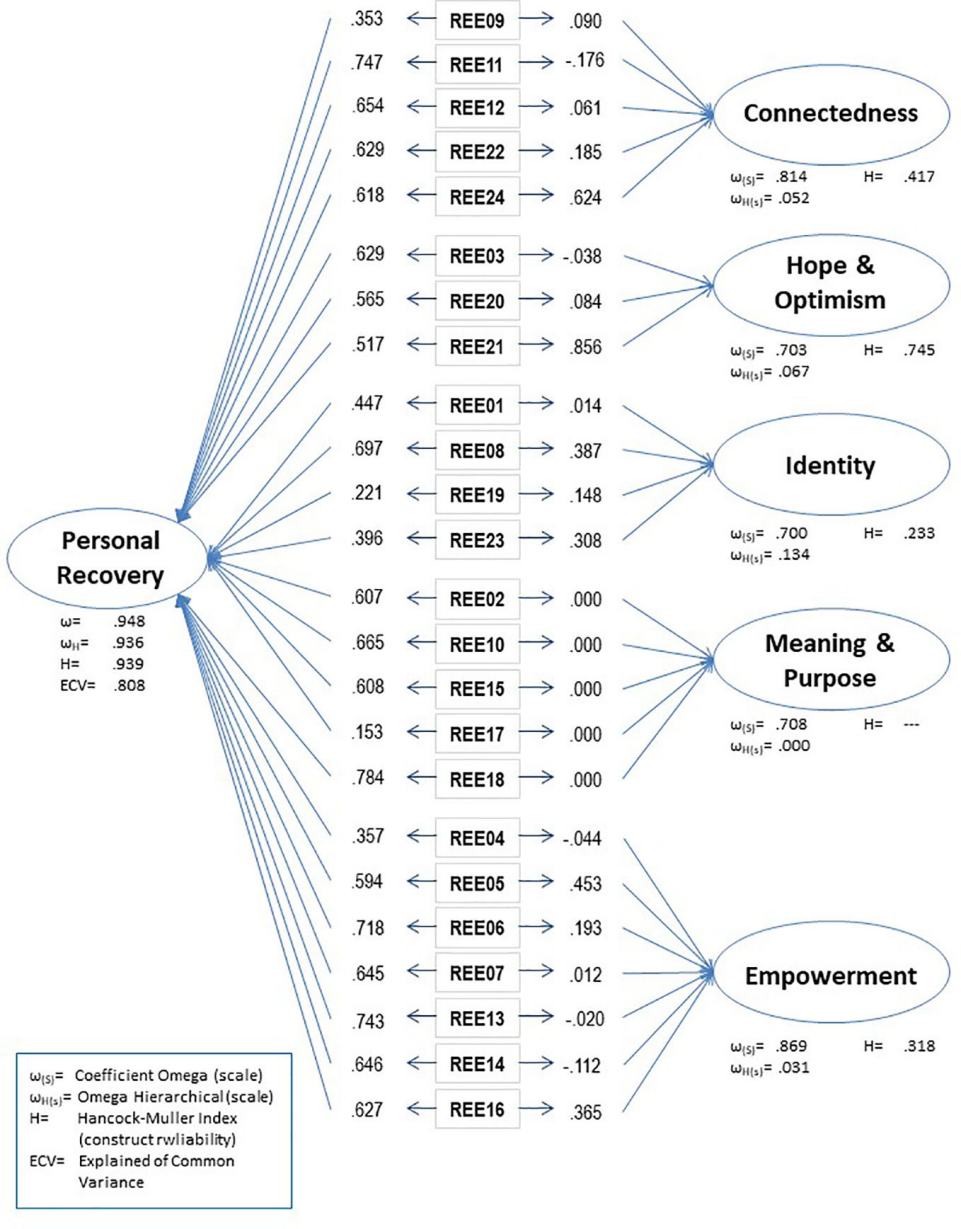
Bi-factor model structure of the REE.

**Figure 2** shows the standardized factor loadings for the bi-factorial model and the coefficients and other statistical indexes. The loadings for the general factor (Personal recovery) ranged from 0.747 to 0.153, with only two items with loadings below 0.350 (I17—*Spirituality—*and I19—*Challenging stigma and discrimination*—). On the other hand, the loadings related to the five domains of CHIME are poor for most items and null in some of them, such as in the Meaning and purpose factor (0.000). Moreover, the reliability *ω* value from the factorial model was 0.948, indicating that a high proportion of the variance was explained by the general factor. Furthermore, *ω*_s_ were relatively high, ranging from 0.869 to 0.700. The *ω*_H_ coefficient was high for the overall score (0.936). This value indicated that 92.6% of the variance of uni-weighted total scores could be attributed to the individual difference on the general factor. The ratio of *ω*_H_ and *ω* (0.936/0.948 = 0.987) indicated that almost all the reliable variance in the total score can be attributed to the general factor; only 1.2% (0.948–0.936) of the reliable variance in the total score can be attributed to the multidimensionality caused by the group factors. In fact, the *ω*_HS_ were very low (ranging from 0.134—Hope and optimism factor—to 0.000—Meaning and purpose—), indicating that subscales scores are not distinct from the general construct the instrument measured.

Finally, the H values were 0.939 (Personal recovery), 0.417 (Connectedness), 0.745 (Hope and optimism), 0.233 (Identity), — (Meaning and Purpose), 0.318 (Empowerment) (**Figure 2**). Reaching the standard criterion of H > 0.70, the general factor and the ‘Hope’ subdomain. The computed ECV is 0.808, indicating that the general factor explains 80.8% of the common variance extracted with 19.2% of the common variance spread across subscales factors. Finally, the PUC value is 0.819; this is, the overwhelming majority of the 276 correlations (24 x 23/2) inform directly to the general factor, which is the target trait the REE instrument was designed to assess.

## Discussion

Recovery is a complex, subjective and multifaceted process (3) that encompasses different domains. However, the CHIME framework (5) has gained greater relevance, providing an overarching model of consensus for researchers and clinicians. In this sense, the present work intended to assess to what extent the REE instrument incorporated the dimensions of the CHIME. First, an expert’s judgement procedure, so as to classify REE items according to the domains of the CHIME framework, was conducted. Second, a factorial procedure to assess user’s responses to the REE according to CHIME was undertaken.

The percentage of agreement between the judges was high (87.50%) and the kappa coefficient for the total score was satisfactory (0.74), and for the five dimensions was moderate (between 0.69 to 0.57) indicating an acceptable convergence in the experts´ responses. In the Hope and optimism dimension, there has been a total agreement in the 3 items that refer to this dimension. The Connectedness dimension has also a high convergence, where only one of the experts has had a divergent judgement with the rest. More heterogeneity resulted in the other three dimensions (Identity, Meaning and Purpose and Empowerment) For example, in item 15—*Having my basic needs met is important to my recovery—*three judges have chosen the same dimension (Meaning and Purpose) and the other two have related this item with other two different dimensions (Identity, and Empowerment respectively), while in Item 6—*Improving my general health and wellness is important to my recovery—*two experts have associated this item with the Meaning and Purpose dimension, and the other three with Empowerment, but assigning it to two different subdimensions.

Furthermore, given the existence of subdimensions in each CHIME domain, rater agreements were higher in assigning items to domains than to subdimensions. For instance, item 21—*Having positive role models is important for my recovery—*is included in Hope and Optimism by all the experts, but three of the judges selected the first subdomain (Belief in the possibility of recovery), another one the third one (Hope-inspiring relationships), and still another expert choose the forth subdimension (Positive thinking and valuing success). Such responses might be indicating that the CHIME subdomains, within each category, are flexible and permeable, allowing for an interaction. As pointed out by Stuart and his colleges (6), there is a dynamic interaction between the CHIME themes, which makes it difficult to clearly delimit them.

Once items’ convergence with those latent variables specified by the experts’ judgment was verified, the global model was quantitatively tested. The unidimensional structure, which was originally proposed, has yielded adequate indexes. The second structure, which conceptually fits the CHIME framework better, suggested five correlated factors and converged satisfactorily.

It can be considered that the second order factor structure (five domains subsumed in a general second order factor of Personal Recovery) could explain better the complexities of this theoretical model. This structure converged adequately, since appropriate indexes have resulted from the CFA. As it can be shown in the visual representation, in the second order structure all the items have acceptable factor loadings in the CHIME dimensions, implying that all items saturate in each of the proposed domains. Hierarchically, these five latent variables (Connectedness, Hope and Optimism, Identity, Meaning and Purpose and Empowerment) have a high weight in the underlying dimension of Personal Recovery. Thus, this structure supports the theoretical model of the CHIME conceptual framework. However, further evidence on the goodness of fit of this model is needed, since in this model one of the indexes has not reached the standard criterion (BB-NFI= 0.807).

Similarly, a bi-factorial model could also account for this framework, since it converged satisfactorily and obtained reasonable indexes; however, the items loadings suggested a different scenario: a principal factor assessing Personal Recovery, with high factor loadings for all the items, except for five of them that have loadings below .40 and also high loadings in the five dimensions of the CHIME. The bifactorial indexes (19, 22) suggested that the multidimensional model is not adequate, since it is indicating that unidimensionality is the best solution to assess Personal Recovery.

In sum, the data from the field study fitted the proposed dimensional structure alternatives, although it has been found that the more complex models (second-order factorial and bifactorial models) are the ones with the best fit values. The unifactorial and bifactorial models lead us to conclude that is mathematically more feasible, to consider a single factor that would explain the ‘Recovery’ construct. This solution is undoubtedly the most parsimonious and it would lead to consider that the 24 elements of the REE constitute an adequate indicator of recovery. In this regard, accepting the uni-dimensionality of the REE implies that the instrument does not differentiate the varied nuances or dimensions that make up the recovery construct. However, the classification of the REE items conducted by the raters with respect to the proposed classification of the CHIME model is indicating that accepting this dimensionality in the REE could be also appropriate. In the same way, when this classification is tested by means of structural equation techniques—in a five correlated factors model or in one of five subsumed factors in a general factor—the data confirmed those conceptual models. Thus, the debate should be centered in the criterion to be used: theoretical or statistical. In the specific case of the recovery concept in chronic mental illness, where the debate is ongoing and there is no widely accepted consensus, we believe that it would be a priority to follow the theoretical approach so as to consolidate the model with further evidences. The results of this study suggest the REE can be an instrument for assessing personal recovery that, simultaneously, allows to create a single indicator based on the 24 items contribution, and at the same time, it makes it possible to differentiate the five specific dimensions in which is based the CHIME recovery model.

The main limitation of this study is that the sample, despite being representative of the Mental Health Services of Biscay, it is also idiosyncratic of a particular geographic area of Spain. This fact could be determining the way services users are experiencing their recovery process. Thus, a possible future line of research could be to replicate this in a different culture or country. For future studies it would be interesting to study further the psychometric properties such as sensibility to change and predictive validity.

In sum, the present study goes beyond the first REE proposal offering evidence of a five dimensional structure based on the CHIME theoretical framework. However, problems in the conceptualization of the recovery concept, and consequently in the way it is measured remain. Notwithstanding, efforts made to carry out a classification and enumeration of the different characteristics are valuable such as the INSPIRE instrument (26), which is an appropriate tool develop from the CHIME framework, of staff support for recovery, and it is composed by two subscales: 20-items support subscale and 7-items relationships subscale.

To conclude, the present study has verified that REE can be a valid instrument to verify the CHIME conceptual framework so as to conceptualize Personal recovery, since two of the models tested (bi-factorial and the second order factor structures) have resulted in adequate indexes and theoretical explanations. Moreover, the REE could be an interesting instrument since it not only measures the relative importance that the service users give to the different elements related to recovery as it has been presented in the present research, but also measures other variables of interest. REE could also be used to assess the experience that the service users have around those elements in the Mental Health Services, which allows measuring the gap between what is value as important by users and what is their perceived experience in terms of those 24 recovery elements.

## Data Availability Statement

The datasets generated for this study are available on request to the corresponding author.

## Ethics Statement

The studies involving human participants were reviewed and approved by Clinical Research Ethics Committee of the Health Services of the Basque Country. The patients/participants provided their written informed consent to participate in this study.

## Author Contributions

PP was a major contributor in writing the manuscript and analyzing the data. JU is the Principal Researcher of the project and has contributed in all the process. SG has contributed in the design and revised all the manuscript. MM-C has participated in the sample collection and revised the manuscript. PR is the author of the instrument and has revised the manuscript. II was the major contributor analyzing the data and writing the manuscript. All authors contributed to the article and approved the submitted version.

## Funding

This research was funded by the Department of Health of the Basque Government (2013111088). In addition, the author PP is a beneficiary of a Predoctoral research scholarship given by the Basque Government (PRE_2017_2_0179).

## Conflict of Interest

The authors declare that the research was conducted in the absence of any commercial or financial relationships that could be construed as a potential conflict of interest.
